# Cognitive Restraint and History of Dieting Are Negatively Associated with Organic Food Consumption in a Large Population-Based Sample of Organic Food Consumers

**DOI:** 10.3390/nu11102468

**Published:** 2019-10-15

**Authors:** Kelly Virecoulon Giudici, Julia Baudry, Caroline Méjean, Denis Lairon, Marc Bénard, Serge Hercberg, France Bellisle, Emmanuelle Kesse-Guyot, Sandrine Péneau

**Affiliations:** 1 Equipe de Recherche en Epidémiologie Nutritionnelle (EREN), Centre de Recherche en Epidémiologies et Biostatistiques, Inserm (U1153), INRA, CNAM, Université Paris 13, Sorbonne Paris Cité, F-93017 Bobigny, France; j.baudry@eren.smbh.univ-paris13.fr (J.B.); caroline.mejean@inra.fr (C.M.); m.benard@eren.smbh.univ-paris13.fr (M.B.); s.hercberg@eren.smbh.univ-paris13.fr (S.H.); f.bellisle@uren.smbh.univ-paris13.fr (F.B.); e.kesse@eren.smbh.univ-paris13.fr (E.K.-G.); s.peneau@uren.smbh.univ-paris13.fr (S.P.); 2 INRA, UMR 1110, Marchés, organisations, institutions et stratégies d’acteurs (MOISA), F-34000 Montpellier, France; 3 Nutrition, obésité et risque thrombotique (NORT), Inserm, UMR S 1062, Aix Marseille Université, INRA 1260, F-13385 Marseille, France; denis.lairon@orange.fr; 4 Santé Publique France, Unité de Surveillance et d’Épidémiologie Nutritionnelle (USEN), Université Paris 13, Sorbonne Paris Cité, F-93017 Bobigny, France; 5 Département de Santé Publique, Hôpital Avicenne, F-93000 Bobigny, France

**Keywords:** cognitive restraint, dieting, organic food, eating behavior, food intake

## Abstract

Organic food consumption has risen in many countries during the past decades, but individual motives leading to these choices remain unclear. This study aimed to evaluate the associations between cognitive restraint (CR), history of dieting and organic food intake. This cross-sectional analysis included 20,085 organic food consumers from the NutriNet-Santé cohort. CR (range score 1–4) was evaluated by the Three-Factor-Eating-Questionnaire and practice of dieting (never vs. past/current) was assessed by an ad hoc questionnaire. Frequencies of organic food intake overall and in 16 food groups were assessed by the Organic Food Frequency Questionnaire. Linear regression and analysis of covariance (ANCOVA) were performed to investigate the association between CR score, history of dieting and contribution of organic food intake to the total food intake. A lower overall contribution of organic options in the diet was observed in women with higher levels of CR (β = −3.61%, 95% CI: −4.32; −2.91 for 1 point of CR, *p* < 0.001) and with a history of dieting (31.1 ± 0.4% in past/current vs. 32.6 ± 0.3% in never dieters; *p* = 0.001). Consistent associations were observed in men with a history of dieting (26.4 ± 0.8% in past/current vs. 28.7 ± 0.4% in never dieters; *p* = 0.012). Overall, individuals—in particular women—with higher CR scores or with a history of dieting selected fewer organic food options. Our findings illustrate the complexity of potentially concurrent motives to food choices, in a context of increasing interest in organic food consumption.

## 1. Introduction

Foods from organic farming are produced in alternative systems that prohibit the use of synthetic chemical fertilizers, pesticides or the practice of intensive animal husbandry and therefore exert lower environmental impact compared with traditionally grown products [1]. Organic standards may vary according to the region but, as accepted by the European Union, organic products are those made without the use of synthetic pesticides and artificial fertilizers; without the use of growth hormones and antibiotics in livestock production (a minimum usage of antibiotics is admitted in very specific cases and is strictly regulated), without genetically modified organisms (GMOs) and their derived products; foods containing at least 95% of organic ingredients [1]. Various potential benefits of organic food on human health have been suggested including reduced risk of allergic diseases [2], overweight and obesity [2] and cancer [3]. In 2019, the French national dietary guidelines have integrated the mode of production, and in particular the importance of selecting organic options, in their recommendations [4]. According to the French Agency for the Development and Promotion of Organic Agriculture [5], in 2017 expenses for organic food consumption at home in France corresponded to 4.4% of total expenses for general food consumption, an increase of 18% compared to 2016.

Since the number of consumers of organic food is rising, it is of increasing importance to understand the underlying reasons and motivations associated with such choices. Health and ethics/environmental concerns have been shown to be important drivers of food choices in organic food consumers [6]. However, to our knowledge, potential weight-related motives have not been investigated in association with organic food consumption. Cognitive restraint and dieting for weight control purposes are commonly observed [7,8,9] due to pervasive pro-dieting messages within the Western world [10]. Cognitive restraint and dieting, although related, are distinct concepts [11].

Cognitive restraint is defined as the control over food intake in order to influence body weight and body shape [12] and exerts quantitative and qualitative influence on dietary intake. In particular, people with higher levels of cognitive restraint have been shown to have a greater intake of healthy food groups [13], to consume less energy [14,15,16], less fat [15,16,17], less carbohydrate [16], and to eat less food in general [14]. The relationship between cognitive restraint and body weight remains controversial: cognitive restraint was associated with higher body mass index (BMI) or other adiposity measures in some studies [18,19], whereas others reported the opposite [20,21,22] or mixed results [23,24]. It remains unknown, however, whether subjects with high cognitive restraint also take other aspects of food quality into account, such as organic production.

The habit of dieting for weight control is closely associated with cognitive restraint [25]. Weight loss diets generally include energy restriction, but consuming food supplements and rebalancing the proportion of macronutrients (low-carb versus low-fat diets) are also strategies considered by dieters [9,26]. As for cognitive restraint, there is no evidence available indicating whether dieters take other aspects of food quality into account and therefore may have a greater intake of organic food.

Thus, the aim of this study was to evaluate the associations between cognitive restraint, history of dieting and organic food consumption in a large sample of adults participating in the NutriNet-Santé study.

## 2. Methods

### 2.1. Study Design and Population

Participants of this cross-sectional study were volunteers from the NutriNet-Santé study, an ongoing web-based prospective observational cohort that aims to investigate the relationship between nutrition and health, as well as the determinants of dietary patterns and nutritional status. The study was launched in May 2009 in France. The design and the methodology of the study have been described in detail elsewhere [27]. Volunteers were recruited via vast multimedia campaigns and through both traditional and online strategies. Individuals older than 18 years, with Internet access and residency in France were eligible for recruitment. At inclusion, participants had to complete several self-reported web-based questionnaires to assess their diet, physical activity, anthropometric measures, lifestyle characteristics, socioeconomic conditions and health status. Participants then completed this same set of questionnaires every year after inclusion. Every month, sets of optional questionnaires related to determinants of eating behaviors, nutritional status, and specific health-related aspects were sent to every participant.

### 2.2. Ethics, Consent and Permissions

The present study was conducted according to the guidelines laid down in the Declaration of Helsinki. The NutriNet-Santé study was approved by the Institutional Review Board of the French Institute for Health and Medical Research (IRB Inserm no. 0000388FWA00005831) and the ‘Commission Nationale de l’Informatique et des Libertés’ (CNIL no. 908450 and no. 909216). All subjects signed an electronic informed consent. The NutriNet-Santé Study was previously registered in the European Clinical Trials Database (EudraCT, eudract.ema.europa.eu) as 2013-000929-31.

### 2.3. Data Collection

#### 2.3.1. Cognitive Restraint

Cognitive restraint was assessed through an optional questionnaire, 14 months after baseline assessment (between July 2010 and January 2011 for most participants). The French version of the revised 21-item Three-Factor Eating Questionnaire (TFEQ-R21) [28], from which 6 items cover cognitive restraint aspects, was used. Questions assessed the control over food intake in order to influence body weight and body shape, (e.g., “I consciously hold back at meals to keep from gaining weight.”), and answers were provided using 4-point scales (definitely true, mostly true, mostly false and definitely false). Cognitive restraint raw scores (range 6–24) were transformed to a 1–4 scale in which higher scores indicated higher levels of cognitive restraint. Internal consistency (Cronbach’s α coefficient) of the cognitive restraint items was 0.79 in our sample.

#### 2.3.2. History of Dieting

Information regarding the history of dieting reported in 2014 (between May and November for most participants) was collected using a self-administered questionnaire. Specifically, participants were asked the following questions: “Are you currently dieting in order to lose weight?” and “In the past, have you been dieting in order to lose weight?” Participants with positive answers to at least one of those questions were identified as dieters (either in the past or presently). Subjects with negative answers to both questions were identified as never dieters.

#### 2.3.3. Organic Food Intake Data

In 2014 (between June and October), participants of the NutriNet-Santé cohort were invited to complete an optional semi-quantitative Food Frequency Questionnaire focusing on organic food products (Org-FFQ), containing 264 food and beverage items. The Org-FFQ was based on a previously validated FFQ [29] supplemented by a section pertaining to the frequency of organic food intake. The questionnaire has been described elsewhere [30]. Briefly, subjects were asked to report their frequency and quantity of consumption over the past year for each of the 264 items and to estimate the frequency of organic food intake for each food (with the question: “How often was the product of organic origin?”). Five-point ordinal items were used (never, rarely, half the time, often and always). The term ‘organic’ referred to European Union- and France-certified organic products. Organic food intake frequency was computed for each food group by applying a weight of 0, 0.25, 0.5, 0.75 and 1 to the five respective categories of frequency. Organic consumers of a determined food group were defined as those who reported consuming at least “rarely” one of the organic food items of this group.

Beverage and food items were aggregated into 16 food groups: fruit and vegetables (including juices and soups); seafood; red meat, poultry and processed meat; eggs; dairy products (milk and yogurt); dairy and meat substitutes (mostly soy based products); starchy refined foods; whole grains; legumes; fast food (pizzas, sandwiches, quiches, pies); snacks (chips, salted biscuits); fatty sweets (including cake, chocolate, ice cream, pancakes); non-fatty sweets (including honey, jelly, sugar, candy); fats (oils, butter and margarine); non-alcoholic beverages; alcoholic beverages.

Basal metabolic rate (BMR) was estimated by Schofield equations [31] according to sex, age, weight and height collected at enrollment in the study. Energy requirement, accounting for physical activity level (set by default at 1.55) and BMR, was compared with energy intake. The ratio between energy intake and energy requirement was calculated. Subjects with ratios below 0.35 or above 1.93 (previously identified cutoffs) in the FFQ were considered as having an implausible energy intake and were removed from the analyses.

Mean time between answering the TFEQ-R21 and the Org-FFQ was 30.9 months, and mean time between the information regarding the history of dieting and the Org-FFQ was 1.5 months.

#### 2.3.4. Sociodemographic, Anthropometric and Lifestyle Characteristics

Self-administered questionnaires were used to collect data on sociodemographic, anthropometric and lifestyle characteristics in 2014 (between May and November for most participants). Age, sex, educational level (primary; secondary; under graduate; post graduate), last occupation (managerial staff or intellectual profession; intermediate professions; self-employed or farmer; employee or manual worker; never employed), monthly household income (<1200 euros; 1200–1799 euros; 1800–2700 euros; >2700 euros; refused to declare), urban unit size (rural community, urban <20,000 habitants; urban 20,000–200,000 habitants; urban >200,000 habitants), family situation (living with a partner without children; living with a partner with children; single without children; single with children), physical activity level (low; intermediate; high), declared height and weight were recorded.

More precisely, monthly income per household unit was calculated by converting the number of people of the household into several consumption units (CU) according to a weighting system proposed by the National Institute of Statistics and Economic Studies (Institut National de la Statistique et des Études Économiques—INSEE): one CU is attributed for the first adult in the household, 0.5 for other persons aged 14 or older and 0.3 for children under 14 years old [32]. Physical activity level was declared by participants by using a short form of the French version of the International Physical Activity questionnaire [33], which was self-administered online. The weekly energy expenditure expressed in metabolic equivalent task minutes per week was estimated, and 3 categories of physical activity were constituted (low (<30 min/day), moderate (30–59 min/day) and high (≥60 min/day)). Self-reported height and weight data collected in 2014 were used. BBMI was calculated as the ratio of weight (kg) to the square of height (m^2^). Subjects were classified as underweight or normal weight (BMI < 25 kg/m^2^), overweight (BMI from 25 to 29.9 kg/m^2^) or obese (BMI ≥ 30 kg/m^2^) according to World Health Organization [34] reference values.

An a priori nutritional diet quality score, the modified ‘French National Nutrition and Health Program’ (Programme National Nutrition Santé—PNNS) Guidelines Score (mPNNS-GS)—which reflects the adherence to the French nutritional recommendations [35] was also calculated. Briefly, the score has a range of 0 to 13.5 points, with a higher score indicating a better overall nutritional quality of the diet. The score includes 12 components: eight refer to food serving recommendations and four refer to moderation of nutrients or food. Points are deducted for overconsumption of salt and sweets, and also when energy intake exceeds the necessary energy level by more than 5%.

### 2.4. Statistical Analysis

From the 112,468 participants of the NutriNet-Santé cohort that received the Org-FFQ, 37,685 participants completed the questionnaire. Within this sample, we included individuals with no missing covariates (*n* = 37,305), who were not detected as under- or over-reporters (*n* = 35,196), and not living overseas (*n* = 34,453). Among them, we selected those who had completed the TFEQ-R21 (*n* = 21,516). From this group, only organic food consumers were retained in the present study (93.3%), leading to a final sample of 20,085 subjects.

Characteristics of subjects were presented as frequencies for categorical variables and means and standard deviations for continuous variables. Comparisons between included and excluded subjects (those who received the Org-FFQ but were not included in our final sample) were performed using chi-square tests for categorical variables and Mann–Whitney U tests for continuous variables, as appropriate. All subsequent analyses were performed separately for men and women given the differences in the practice of dieting between men and women [36] and in cognitive restraint [23]. Sex*cognitive restraint interactions were non-significant for all food groups apart from a significant interaction for organic food overall (*p* = 0.02) and for the fatty sweet group (*p* = 0.03). In addition, none of the sex*history of dieting interactions were significant. Cognitive restraint level, history of dieting and overall mean percentage of organic food intake according to sociodemographic and lifestyle characteristics were described. Bivariate analyses were performed using chi-square, Pearson correlations, Student, Mann–Whitney U and Kruskal–Wallis tests, as appropriate. When conditions for parametric tests were not met, non-parametric tests were performed.

Linear regression analyses were performed with cognitive restraint as an independent variable and contribution of organic food intake to the total food intake as the dependent variable. In addition, analysis of covariance (ANCOVA) models were performed with history of dieting as an independent variable and contribution of organic food intake to the total food intake as the dependent variable. Adjusted means of proportions of the contribution of organic food intake to the total food intake were calculated and proportions were compared across categories of history of dieting for the 16 food groups. Potential confounders were identified in the literature and selected based on a directed acyclic graph (DAG) (Supplementary Figure S1). A first model was adjusted for age, educational level, occupational categories, monthly household income, urban unit size and family situation (potential confounders that do not appear likely to be intermediate variables). A second model was additionally adjusted for physical activity level, energy intake, mPNNS-GS, total intake of the food group and BMI (including potential confounders that could also be on the causal pathway). Assumptions for linear regression models were verified. All analyses were performed using SAS software (version 9.3; SAS Institute Inc., Cary, NC, USA). Statistical tests were two-sided, and significance was set at 5%.

## 3. Results

### 3.1. Characteristics of the Sample

Included volunteers were older (55.2 years, SD = 13.7 vs. 45.3 years, SD = 14.4), belonged to a higher-level occupational category (managerial staff or intellectual profession) (38.2% vs. 30.9%) and were less often never employed (6.5% vs. 13.5%), declared higher household income (>2700 euros) (31.0% vs. 23.0%), and more often reported high physical activity level (37.5% vs. 32.1%) compared with excluded subjects (all *p* < 0.001). No difference was observed for education level and BMI.

Cognitive restraint level and history of dieting were compared according to sociodemographic and lifestyle characteristics in both sexes (Table 1). Women and men with higher CR level were older, had a lower education level, higher income, higher physical activity level, BMI and diet quality, and lived more often with a partner without children. In addition, in women specifically, CR level was greater in “self-employed, farmer” professional categories. Women who were past/current dieters were younger, more often employee or manual workers, less physical active, had a lower income, a lower education level, a higher BMI and a greater diet quality compared with those who never dieted. Men who were past/current dieters were younger, less physical active, had a lower income and a higher BMI. In addition, 85.8% of women and 77.7% of men were considered past dieters, while 14.2% of women and 22.3% of men were current dieters. Mean percentage of overall organic food intake out of total food intake was compared according to sociodemographic and lifestyle characteristics in both sexes (Table 2). Women with higher contribution of organic food out of total food intake were older, had greater education levels, income, physical activity level, diet quality and lower BMI. In addition, they belonged more often to the “self-employed, farmer” professional category and lived in smaller cities. Men with higher contribution of organic food were younger, more often “self-employed, farmer” and “never employed”, lived in smaller cities, and had higher physical activity level, higher diet quality and lower BMI.

### 3.2. Cognitive Restraint and Organic Food Intake

Table 3 shows multivariable linear regression analyses between CR score and organic food intake out of total food intake, among organic food consumers. Among women, higher level of cognitive restraint was associated with lower contribution of organic food choices (from total consumed food) overall and specifically for all 16 food groups, for both adjustment models (*p* < 0.001 for all). Among men, higher level of cognitive restraint was associated with lower contribution of organic fruit and vegetables, dairy and meat substitutes, whole grains, legumes, fast food, snacks, non-fatty sweets, fats and non-alcoholic beverages in Model 1. After additional adjustments for physical activity level, energy intake, mPNNS-GS, total intake of the food group and BMI, associations for the whole grain group did not remain significant. Findings for the group of red meat, poultry, processed meat were significant in the second model only. No association was observed when considering all foods together, and for the specific food groups: seafood, eggs, dairy products, starchy refined foods, fatty sweets, and alcoholic beverages.

### 3.3. History of Weight Loss Diet and Organic Food Intake

Table 4 shows the proportions of organic food consumed out of total intake across categories of dieting, among organic food consumers. In the first model, women who were past or current dieters had lower frequencies of organic food choices overall and of fruits and vegetables, dairy products, dairy and meat substitutes, starchy refined foods, whole grains, legumes, fast food, snacks, fatty sweets, non-fatty sweets, fats, non-alcoholic beverages and alcoholic beverages, compared to women who had never been on a diet. No association was observed for the remaining food groups. After additional adjustments, results for the groups of fruits and vegetables, fast food, fats and alcoholic beverages did not persist significant. Men who were past or current dieters presented lower mean frequencies of organic food choices in the overall diet (all groups) and for fruits and vegetables, eggs, starchy refined foods, fast food, fatty sweets, non-fatty sweets and non-alcoholic beverages (in Model 1), but only associations for the overall diet remained significant after additional adjustments (Model 2).

## 4. Discussion

In this study, contribution of organic options overall and from various food groups consumed in the previous year was examined in men and women as a function of cognitive restraint level and history of dieting. Women with higher levels of cognitive restraint or with a history of dieting reported a lower contribution of organic foods (overall and for most food groups) in their diet. Consistently, men with a history of dieting reported a lower contribution of organic food (overall and for specific food groups), while the association with CR level was observed for specific food groups only.

Compared with other studies, absolute mean scores of cognitive restraint observed in our population were similar [37] or higher [24]. By contrast, absolute frequencies of history of dieting were lower than reported in a recent meta-analysis [9]. The proportion of organic food consumers in our sample, defined as consumers reporting at least one instance of intake of one organic food item over the past year, was higher compared with previous studies from Denmark [38], Norway [39] and Germany [40]. Such differences may be due to the type of population considered (age and country) or the methodology used.

To our knowledge, the associations between cognitive restraint and organic food intake have not been explored so far in the literature. Cognitive restraint has been shown to be associated with healthier food choices [13], lower energy intake [14,15,16], and lower fat and carbohydrate intake [15,16,17]. Similarly, there are to our knowledge no data available about an association between dieting and organic food intake, although weight loss strategies focus on various aspects of the diet including a control on both quantity and quality of the food [9,41].

Our results suggest that individuals with cognitive restraint or with a history of dieting might be less interested in characteristics of foods not specifically related to energy or macronutrient, such as the production mode. It is possible that restrained individuals are more self-centered and therefore score lower on motives predicting organic intake that not only include self-centered motives (such as health) but also altruistic motives (such as environmental) [42]. However, there are no data in the literature supporting this hypothesis. The few data available on personally traits indicate that individuals with greater cognitive restraint have higher level of conscientiousness [43,44] and neuroticism [44] while no association with other traits such as openness [43,44] was observed.

Another hypothesis to account for this difference is that focusing on both weight control and organic intake at the same time could be too complex or time consuming for participants to do it in a daily routine. People engage daily in multiple eating episodes [45,46], in a sequence of processes that involves interrelated decisions incorporating a great variety of food behaviors [47]. As previously mentioned, people with higher levels of cognitive restraint and/or on weight loss diets focus on nutritional aspects of the diet including energy and macronutrients distribution [13,14,15,16,17]. On the other hand, individuals concerned about consuming organic food tend to focus on other health and/or environmental characteristics of the products [48,49,50], i.e., sustainable agriculture, no use of agrochemicals such as synthetic fertilizers and pesticides, no use of genetically modified organisms [1], and show higher interest in vegetarian or vegan diets [50,51]. It has been shown in studies evaluating nutrition information on food labels that people have difficulty in processing much information at the same time [52]. Other studies also confirm the complexity of focusing on many dietary aspects when planning one’s diets [53,54,55].

The cost of food is another aspect that may partly explain our results, given its influence on diet choices [56]. Consumption of low-fat and/or calorie-reduced food is part of several weight loss strategies. In addition, individuals with higher levels of cognitive restraint have been shown to consume a larger amount of low-fat and calorie-reduced foods [17]. These type of diet foods tend to be more expensive, compared to their regular versions and to other energy-dense products [57,58]. Considering that organic food also has a higher cost [59], it is possible that individuals with greater concern for their body weight will prioritize this aspect over an organic claim. Moreover, it should be noted that the offer of processed foods that are both organic and reduced in energy, fat or sugar compared to their regular versions (i.e., diet foods) is scarce, possibly forcing individuals to choose between one or the other claim.

Cognitive restraint was higher in women than in men. In addition, associations between cognitive restraint levels and organic food consumption were stronger among women. Such results, added to the fact that women are more aware of body image and eating habits than men [60], could suggest that, independent of the level of cognitive restraint, the behavior associated with restraint is more marked in women, leading to differences in organic food choices. A similar interpretation can be made in the case of dieting.

### Strengths and Limitations

Our study included a large sample size, which allowed for high precision estimates. In addition, having a sample with subjects representing various sociodemographic characteristics and weight status allowed controlling for confounding factors while keeping an adequate statistical power. However, it should be noted that other potential confounders (such as health concerns) were not addressed. In addition, it is possible that certain variables (particularly physical activity, energy intake, mPNNS-GS or BMI) could have a mediating or modification effect on the association between cognitive restraint/history of dieting and organic food intake. Participants were volunteers, and thus more likely to be interested in nutritional topics including weight control, sustainable food issues, and healthy lifestyle than the general population, which may create some recruitment biases. They are mainly mature adults, with a relatively high level of education and income, which have been associated with greater sustainable consumption [61,62]. In addition, no weighting scheme was implemented to take into account potential differences between our population and the general French population. Caution is therefore needed when generalizing the results. The semi-quantitative food frequency questionnaire used in this study included a very large range of foods (264 items). Of note, questions concerning the frequency of organic food consumption have not been validated. Organic food consumption was obtained by attributing the percentages 0, 25, 50, 75, and 100, to the modalities “never”, “rarely”, “half the time”, “often” and “always”. A possible overestimation of frequencies of organic food choices in the present sample cannot be discarded. It must be noted that the proportion of organic food consumers was not substantially modified in a sensitivity analysis assessing the robustness of the scale, whose details have been published elsewhere [30]. In addition, considering that the completion of the Org-FFQ was optional and given the long set of items of the questionnaire, participants with high sustainable food concerns could be more likely to complete it compared to other participants of the cohort.

Another limitation of the study pertains to its cross-sectional design that does not enable the demonstration of causality or the examination of temporal variations in the measured variables. A large number of tests have been performed, which amplifies the probability of a false-positive finding. However, since these analyses are exploratory, multiple adjustment was not performed as recommended [63]. Cognitive restraint was assessed 30 months before the FFQ assessing organic food consumption. Although restraint is a relatively stable individual trait [64], it has been shown to increase as a result of dieting [65], so the level of restraint at the time of completing the Org-FFQ may have changed, particularly in dieters. Finally, data were obtained from self-report questionnaires, which are prone to measurement error, as also was height and weight information. However, self-reported anthropometric data are reliable for the identification of relations in epidemiologic studies [66] and have been validated in the NutriNet-Santé cohort [67].

## 5. Conclusions

We have shown that cognitive restraint and history of dieting are associated with lower organic food intake among women and to a lower extent among men, in a very large adult cohort. Our study provides new insights into the association between weight-related behaviors and organic food choices. These results are particularly important in a context where the French national dietary guidelines take into account the nutritional quality of food but also its organic production. Further work exploring these associations across populations with different socioeconomic characteristics and levels of organic food intake is needed to extend our knowledge and set up effective nutrition policies.

## Figures and Tables

**Table 1:** Cognitive restraint level and history of dieting according to sociodemographic and lifestyle characteristics in 20,085 organic food consumers (NutriNet-Santé study (2010–2014)).

	Total (*n* = 20,085)	Women (*n* = 14,955)					Men (*n* = 5130)				
		Cognitive Restraint	*p*	Never Dieting	Past/Current Diet	*p*	Cognitive Restraint	*p*	Never Dieting	Past/Current Diet	*p*
All		2.33 ± 0.60 ^1^		66.0 ^2^	34.0	**<0.001** ^3^	2.17 ± 0.59		76.7	23.3	**<0.001**
Age (years)	55.2 ± 13.7	0.14 (0.12;0.15) ^4^	**<0.001** ^5^	53.7 ± 13.8	53.1 ± 13.4	**0.006** ^6^	0.16 (0.15;0.18)	**<0.001**	60.4 ± 12.6	59.0 ± 12.4	**<0.001**
Educational level (%)			**<0.001**			**<0.001** ^7^		**0.02**			
Post graduate	34.3	2.26 ± 0.61		68.6	31.4		2.16 ± 0.59		77.1	22.9	0.25
Under graduate	29.3	2.33 ± 0.61		65.8	34.2		2.15 ± 0.61		77.1	22.9	
Secondary	33.5	2.40 ± 0.61		64.1	35.9		2.19 ± 0.59		76.7	23.3	
Primary	2.8	2.41 ± 0.64		59.7	40.3		2.25 ± 0.64		69.0	31.0	
Occupational categories (%)			**0.003**			**<0.001**		0.073			0.06
Managerial staff, intellectual profession	38.2	2.33 ± 0.60		67.6	32.4		2.18 ± 0.59		77.4	22.6	
Intermediate professions	27.5	2.33 ± 0.61		67.8	32.2		2.18 ± 0.59		76.9	23.1	
Self-employed, farmer	3.3	2.39 ± 0.64		66.0	34.0		2.19 ± 0.66		74.9	25.1	
Employee, manual worker	24.6	2.35 ± 0.62		62.8	37.2		2.15 ± 0.59		74.4	25.6	
Never employed	6.5	2.29 ± 0.66		64.8	35.2		1.99 ± 0.66		75.3	24.7	
Monthly household income (%)			**<0.001**			**<0.001**		**0.002**			**0.003**
>2700 euros	31.0	2.36 ± 0.59		68.9	31.1		2.19 ± 0.59		77.6	22.4	
1800–2700 euros	25.6	2.33 ± 0.61		67.0	33.0		2.17 ± 0.59		79.0	21.0	
1200–1799 euros	21.2	2.32 ± 0.62		63.1	36.9		2.18 ± 0.61		73.7	26.3	
<1200 euros	10.1	2.29 ± 0.66		62.6	37.4		2.05 ± 0.61		72.7	27.3	
Refused to declare	12.2	2.34 ± 0.61		66.1	33.9		2.19 ± 0.58		75.7	24.3	
Urban unit size (%)			0.15			0.33		0.32			0.34
>200,000 habitants	44.0	2.34 ± 0.61		65.4	34.6		2.16 ± 0.60		76.0	24.0	
20,000–200,000 habitants	18.1	2.34 ± 0.60		66.5	33.5		2.17 ± 0.59		77.7	22.3	
<20,000 habitants	15.6	2.31 ± 0.63		67.6	32.4		2.21 ± 0.58		75.7	24.3	
Rural community	22.3	2.32 ± 0.62		65.9	34.1		2.16 ± 0.61		77.9	22.1	
Family situation (%)			**<0.001**			0.88		**<0.001**			0.28
With partner without children	57.3	2.37 ± 0.60		67.1	32.9		2.20 ± 0.59		78.2	21.8	
With partner with children	17.6	2.24 ± 0.60		64.1	35.9		2.05 ± 0.57		73.7	26.3	
Single without children	22.8	2.30 ± 0.63		65.6	34.4		2.16 ± 0.63		73.5	26.5	
Single with children	2.3	2.30 ± 0.62		63.0	37.0		2.14 ± 0.63		76.9	23.1	
Physical activity level (%)			**<0.001**			**<0.001**		**0.002**			**<0.001**
High	37.5	2.37 ± 0.63		68.0	32.0		2.20 ± 0.60		78.3	21.7	
Intermediate	41.5	2.31 ± 0.61		66.3	33.7		2.16 ± 0.59		77.5	22.5	
Low	21.0	2.30 ± 0.60		62.4	37.6		2.12 ± 0.59		71.3	28.7	
Body mass index (%)			**<0.001**			**<0.001**		**<0.001**			**<0.001**
Obese (≥30 kg/m^2^)	9.2	2.51 ± 0.54		36.3	63.7		2.38 ± 0.52		45.9	54.1	
Overweight (25 to 29.99 kg/m^2^)	23.5	2.51 ± 0.52		47.7	52.3		2.29 ± 0.55		65.9	34.1	
Underweight/normal weight (<25 kg/m^2^)	67.2	2.26 ± 0.63		74.8	25.2		2.06 ± 0.61		89.3	10.7	
mPNNS-GS ^‡^ (0 to 13.5)	8.5 ± 1.7	0.14 (0.13;0.16)	**<0.001**	8.5 ± 1.8	8.7 ± 1.7	**<0.001**	0.15 (0.14;0.17)	**<0.001**	8.6 ± 1.6	8.5 ± 1.6	0.29

^1^ Mean (sd) (all such value); ^2^ Percentages (all such value); ^3^ *p*-value based on chi-square test (all such test); ^4^ Pearson correlation (95% CI) (all such value); ^5^ *p*-value based on Student-test (all such test); ^6^ *p*-value based on Mann–Whitney U test (all such test); ^7^ *p*-value based on Kruskal–Wallis test (all such test). ^‡^ modified ‘French National Nutrition and Health Program’ (Programme National Nutrition Santé–PNNS) Guidelines Score. Bold values indicate *p* < 0.05.

**Table 2:** Mean percentage of overall organic food intake out of total food intake according to sociodemographic and lifestyle characteristics in 20,085 organic food consumers (NutriNet-Santé study (2010–2014)) ^1^.

	Women (*n* = 14,955)		Men (*n* = 5130)	
	Mean % of Organic Food Intake	*p* ^1^	Mean % of Organic Food Intake	*p* ^1^
All	32.22 (26.84)		28.24 (26.09)	
Age (years)	0.07 (0.06;0.09) ^2^	**<0.001** ^3^	−0.09 (−0.10;−0.07)	**<0.001**
Educational level (%)		**<0.001** ^4^		0.10
Post graduate	34.0 ± 27.3		28.7 ± 26.3	
Under graduate	32.5 ± 26.8		29.4 ± 26.9	
Secondary	30.5 ± 26.4		27.1 ± 25.2	
Primary	27.9 ± 25.9		26.7 ± 26.9	
Occupational categories (%)		**<0.001**		**0.033**
Managerial staff, intellectual profession	33.5 ± 26.5		27.4 ± 25.6	
Intermediate professions	33.0 ± 27.0		28.2 ± 25.6	
Self-employed, farmer	38.3 ± 29.2		32.2 ± 29.2	
Employee, manual worker	29.6 ± 26.3		29.6 ± 27.2	
Never employed	31.2 ± 27.6		33.8 ± 27.1	
Monthly household income (%)		**<0.001**		0.21
>2700 euros	32.7 ± 26.2		28.1 ± 25.8	
1800–2700 euros	33.1 ± 26.8		28.6 ± 25.9	
1200–1799 euros	31.1 ± 27.1		27.8 ± 25.9	
<1200 euros	31.5 ± 28.1		31.8 ± 30.1	
Refused to declare	31.8 ± 26.8		25.9 ± 24.7	
Urban unit size (%)		**<0.001**		**<0.001**
>200,000 habitants	30.5 ± 26.1		25.9 ± 25.2	
20,000–200,000 habitants	32.1 ± 26.8		29.7 ± 26.7	
<20,000 habitants	33.9 ± 27.4		28.7 ± 26.4	
Rural community	34.6 ± 27.8		31.0 ± 26.7	
Family situation (%)		0.27		0.16
With partner without children	32.4 ± 26.6		28.0 ± 25.7	
With partner with children	31.3 ± 26.4		30.3 ± 27.8	
Single without children	32.5 ± 27.5		26.9 ± 25.5	
Single with children	33.8 ± 29.1		33.4 ± 30.9	
Physical activity level (%)		**<0.001**		**<0.001**
High	34.6 ± 27.4		30.6 ± 27.1	
Intermediate	32.1 ± 26.5		27.8 ± 25.7	
Low	28.7 ± 26.1		23.4 ± 23.6	
Body mass index (%)		**<0.001**		**<0.001**
Obese (≥30 kg/m^2^)	26.4 ± 24.9		21.8 ± 21.4	
Overweight (25 to 29.99 kg/m^2^)	29.0 ± 24.9		24.7 ± 23.9	
Underweight/normal weight (<25 kg/m^2^)	33.8 ± 27.4		31.7 ± 27.7	
mPNNS-GS ^‡^ (0 to 13.5)	0.12 (0.10;0.13)	**<0.001**	0.16 (0.15;0.18)	**<0.001**

^1^ Percentages (all such values); ^2^ Pearson correlation (95% CI) (all such value); ^3^ *p*-value based on Student-test (all such test); ^4^ *p*-value based on Kruskal–Wallis test (all such test). ^‡^ modified ‘French National Nutrition and Health Program’ (Programme National Nutrition Santé–PNNS). Bold values indicate *p* < 0.05.

**Table 3:** Multivariable regression analysis between cognitive restraint score and organic food intake out of total food intake in 20,085 organic food consumers (NutriNet-Santé study 2010–2014).

		Women			Men		
		Cognitive Restraint			Cognitive Restraint		
Organic Food Groups		*n*	β (95% CI) ^4^	*p* ^1^	*n*	β (95% CI) ^4^	*p* ^1^
All groups	M1 ^2^	14,955	−3.28 (−3.98;−2.58)	**<0.001**	5130	−0.67 (−1.88;0.53)	0.27
	M2 ^3^	14,955	−3.61 (−4.32;−2.91)	**<0.001**	5130	−0.74 (−1.95;0.47)	0.23
Fruit and vegetables	M1	13,348	−3.40 (−4.16;−2.65)	**<0.001**	4364	−1.62 (−2.98;−0.27)	**0.02**
	M2	13,348	−3.73 (−4.49;−2.97)	**<0.001**	4364	−1.83 (−3.19;−0.48)	**0.008**
Seafood	M1	7998	−1.82 (−2.70;−0.96)	**<0.001**	2571	−0.20 (−1.89;1.49)	0.82
	M2	7998	−2.02 (−2.91;−1.13)	**<0.001**	2571	−0.80 (−2.54;0.95)	0.37
Red meat, poultry, processed meat	M1	11,483	−1.25 (−1.97;−0.53)	**<0.001**	3807	−0.87 (−2.15;0.40)	0.18
	M2	11,483	−1.30 (−2.03;−0.57)	**<0.001**	3807	−1.33 (−2.62;−0.03)	**0.045**
Eggs	M1	12,152	−1.46 (−2.32;−0.61)	**<0.001**	3726	−0.62 (−2.29;1.05)	0.47
	M2	12,152	−1.66 (−2.53;−0.79)	**<0.001**	3726	−0.85 (−2.56;0.85)	0.33
Dairy products	M1	9745	−2.13 (−2.95;−1.30)	**<0.001**	3052	−0.99 (−2.46;0.48)	0.19
	M2	9745	−2.47 (−3.31;−1.63)	**<0.001**	3052	−0.95 (−2.44;0.54)	0.21
Dairy and meat substitutes	M1	5840	−3.83 (−4.99;−2.66)	**<0.001**	1380	−3.27 (−5.86;−0.68)	**0.013**
	M2	5840	−3.98 (−5.23;−2.74)	**<0.001**	1380	−3.83 (−6.73;−0.94)	**0.010**
Starchy refined foods	M1	11,885	−2.42 (−3.34;−1.50)	**<0.001**	3714	−1.43 (−3.06;0.20)	0.09
	M2	11,885	−2.54 (−3.47;−1.61)	**<0.001**	3714	−1.51 (−3.15;0.14)	0.072
Whole grains	M1	6968	−3.67 (−4.73;−2.67)	**<0.001**	2068	−2.03 (−3.95;−0.11)	**0.04**
	M2	6968	−4.42 (−5.63;−3.21)	**<0.001**	2068	−2.03 (−4.30;0.24)	0.080
Legumes	M1	8083	−3.92 (−5.00;−2.84)	**<0.001**	2505	−3.19 (−5.16;−1.22)	**0.002**
	M2	8083	−3.47 (−4.56;−2.38)	**<0.001**	2505	−2.60 (−4.59;−0.61)	**0.010**
Fast food	M1	8396	−4.00 (−4.94;−3.06)	**<0.001**	2639	−2.01 (−3.67;−0.32)	**0.02**
	M2	8396	−4.03 (−4.99;−3.07)	**<0.001**	2639	−2.12 (−3.84;−0.40)	**0.015**
Snacks	M1	7217	−3.32 (−4.46;−2.18)	**<0.001**	2182	−2.07 (−4.00;−0.14)	**0.036**
	M2	7217	−3.36 (−4.46;−2.25)	**<0.001**	2182	−1.99 (−3.87;−0.13)	**0.036**
Fatty sweets	M1	11,892	−3.28 (−4.05;−2.51)	**<0.001**	3834	−1.17 (−2.53;0.18)	0.09
	M2	11,892	−3.47 (−4.25;−2.69)	**<0.001**	3834	−1.05 (−2.42;0.31)	0.13
Non-fatty sweets	M1	10,726	−1.80 (−2.76;−0.84)	**<0.001**	3513	−2.35 (−4.08;−0.62)	**0.008**
	M2	10,726	−1.71 (−2.69;−0.74)	**<0.001**	3513	−2.32 (−4.07;−0.57)	**0.009**
Fats	M1	8316	−3.85 (−4.99;−2.70)	**<0.001**	2539	−3.50 (−5.56;−1.45)	**0.001**
	M2	8316	−3.74 (−4.90;−2.58)	**<0.001**	2539	−2.92 (−5.00;−0.82)	**0.006**
Non-alcoholic beverages	M1	10,997	−3.24 (−3.88;−2.59)	**<0.001**	3350	−1.87 (−3.08;−0.67)	**0.002**
	M2	10,997	−2.97 (−3.63;−2.32)	**<0.001**	3350	−1.80 (−3.03;−0.56)	**0.004**
Alcoholic beverages	M1	8554	−1.97 (−2.79;−1.14)	**<0.001**	3048	−0.71 (−2.15;0.73)	0.33
	M2	8554	−2.07 (−2.90;−1.23)	**<0.001**	3048	−0.89 (−2.38;0.59)	0.24

^1^*p*-values based on linear regressions, with cognitive restraint as a continuous independent variable and organic food intake out of total food intake as a dependent variable; ^2^ Model 1 was adjusted by age, educational level, occupational categories, monthly household income, urban unit size and family situation; ^3^ Model 2 was adjusted by age, educational level, occupational categories, monthly household income, urban unit size, family situation, physical activity level, energy intake, mPNNS-GS, total intake of the group and BMI; ^4^ β coefficients of the cognitive restraint effect can be interpreted as change in organic food intake out of total food intake (mean %) per increase of 1 point in the cognitive restraint scale (ranging from 1–4). Bold values indicate *p* < 0.05.

**Table 4:** Mean percentage of organic food intake out of total food intake, according to sex and history of dieting in 20,085 organic food consumers (NutriNet-Santé study 2010–2014).

		Women				Men			
			Never Dieting	Past/Current Diet			Never Dieting	Past/Current Diet	
Organic Food Groups		*n*	Mean (SE)	Mean (SE)	*p* ^1^	*n*	Mean (SE)	Mean (SE)	*p* ^1^
All groups	M1 ^2^	14,955	33.1 (0.3) ^3^	30.4 (0.4)	**<0.001**	5130	29.3 (0.4)	24.5 (0.7)	**<0.001**
	M2 ^4^	14,955	32.6 (0.3)	31.1 (0.4)	**0.001**	5130	28.7 (0.4)	26.4 (0.8)	**0.012**
Fruit and vegetables	M1	13,348	39.7 (0.3)	37.9 (0.4)	**<0.001**	4364	37.5 (0.5)	34.0 (0.9)	**<0.001**
	M2	13,348	39.3 (0.3)	38.4 (0.4)	0.089	4364	36.8 (0.5)	35.6 (0.9)	0.21
Seafood	M1	7998	31.7 (0.3)	30.9 (0.4)	0.16	2571	33.6 (0.6)	33.4 (1.1)	0.84
	M2	7998	31.6 (0.3)	31.0 (0.5)	0.33	2571	33.5 (0.6)	33.2 (1.1)	0.80
Red meat, poultry, processed meat	M1	11,483	32.0 (0.3)	31.7 (0.4)	0.51	3807	30.6 (0.4)	29.5 (0.8)	0.24
	M2	11,483	31.7 (0.3)	32.3 (0.4)	0.29	3807	30.5 (0.4)	29.9 (0.8)	0.53
Eggs	M1	12,152	72.6 (0.6)	71.9 (0.5)	0.21	3726	67.5 (0.6)	64.8 (1.1)	**0.024**
	M2	12,152	72.4 (0.3)	72.1 (0.5)	0.64	3726	67.0 (0.6)	65.7 (1.1)	0.29
Dairy products	M1	9745	32.9 (0.3)	31.4 (0.4)	**0.007**	3052	31.8 (0.5)	29.9 (0.9)	0.07
	M2	9745	32.7 (0.3)	31.5 (0.4)	**0.027**	3052	31.2 (0.5)	31.3 (0.9)	0.91
Dairy and meat substitutes	M1	5840	80.8 (0.5)	77.6 (0.6)	**<0.001**	1380	77.4 (0.9)	74.3 (1.7)	0.12
	M2	5840	83.5 (0.5)	80.5 (0.7)	**<0.001**	1380	81.4 (1.0)	77.2 (1.9)	0.056
Starchy refined foods	M1	11,885	42.7 (0.4)	39.6 (0.5)	**<0.001**	3714	40.3 (0.6)	36.9 (1.0)	**0.003**
	M2	11,885	42.0 (0.4)	40.4 (0.5)	**0.012**	3714	39.3 (0.6)	39.0 (1.1)	0.82
Whole grains	M1	6968	56.8 (0.4)	53.6 (0.6)	**<0.001**	2068	54.5 (0.7)	52.1 (1.2)	0.09
	M2	6968	59.4 (0.5)	57.2 (0.7)	**0.006**	2068	57.0 (0.8)	56.9 (1.5)	0.93
Legumes	M1	8083	60.0 (0.4)	56.7 (0.6)	**<0.001**	2505	54.9 (0.7)	52.3 (1.3)	0.08
	M2	8083	59.3 (0.4)	57.6 (0.6)	**0.020**	2505	53.7 (0.7)	54.7 (1.3)	0.50
Fast food	M1	8396	35.1 (0.4)	33.2 (0.5)	**0.002**	2639	33.7 (0.6)	30.5 (1.1)	**0.008**
	M2	8396	34.6 (0.4)	33.8 (0.5)	0.19	2639	33.2 (0.6)	31.5 (1.1)	0.20
Snacks	M1	7217	46.0 (0.4)	42.8 (0.6)	**<0.001**	2182	40.3 (0.7)	37.9 (1.3)	0.09
	M2	7217	45.3 (0.4)	43.3 (0.6)	**0.010**	2182	39.2 (0.6)	39.8 (1.2)	0.68
Fatty sweets	M1	11,892	31.2 (0.3)	28.4 (0.4)	**<0.001**	3834	29.1 (0.5)	26.9 (0.9)	**0.021**
	M2	11,892	30.8 (0.3)	28.8 (0.4)	**<0.001**	3834	28.3 (0.5)	28.7 (0.9)	0.74
Non-fatty sweets	M1	10,726	56.1 (0.4)	53.0 (0.5)	**<0.001**	3513	54.5 (0.6)	49.8 (1.1)	**<0.001**
	M2	10,726	55.5 (0.4)	53.7 (0.5)	**0.006**	3513	53.4 (0.6)	51.8 (1.2)	0.23
Fats	M1	8316	51.9 (0.4)	49.7 (0.6)	**0.003**	2539	47.8 (0.7)	44.7 (1.3)	0.042
	M2	8316	51.3 (0.4)	50.4 (0.6)	0.24	2539	46.7 (0.7)	47.4 (1.4)	0.69
Non-alcoholic beverages	M1	10,997	26.9 (0.2)	24.5 (0.4)	**<0.001**	3350	26.6 (0.4)	23.7 (0.8)	**0.001**
	M2	10,997	26.4 (0.3)	25.0 (0.4)	**0.002**	3350	26.0 (0.4)	24.9 (0.8)	0.24
Alcoholic beverages	M1	8554	29.5 (0.3)	28.1 (0.4)	**0.008**	3048	30.0 (0.5)	28.1 (0.9)	0.07
	M2	8554	29.3 (0.3)	28.3 (0.4)	0.10	3048	29.8 (0.5)	28.6 (1.0)	0.32

SE, standard error of the mean; ^1^ *p*-value based on analysis of covariance (ANCOVA) with history of dieting as an independent variable and organic food intake out of total food intake as a dependent variable; ^2^ Model 1 was adjusted by age, educational level, occupational categories, monthly household income, urban unit size and family situation; ^3^ Mean percentage of organic food intake out of total food intake; ^4^ Model 2 was adjusted by age, educational level, occupational categories, monthly household income, urban unit size, family situation, physical activity level, energy intake, mPNNS-GS, total intake of the group and BMI. Bold values indicate *p* < 0.05.

## Supplementary Materials

The following are available online at https://www.mdpi.com/2072-6643/11/10/2468/s1, Figure S1. Directed acycly graph (DAG) representing associations between cognitive restrain/history of dieting and organic food consumption. Minimal sufficient adjustment sets for estimating the total effect of cognitive restrain/history of dieting on organic food intake as suggested by the DAG are: age, BMI, dietary intake, family situation, socioeconomic status, urban unit size. Colors of variables: green—exposure; blue—outcome; red—co-variables. (NutriNet-Santé study (2010–2014).

## References

[1] Gomiero T., Pimentel D., Paoletti M.G. (2011). Environmental Impact of Different Agricultural Management Practices: Conventional vs. Organic Agriculture. Crit. Rev. Plant Sci..

[2] Mie A., Andersen H.R., Gunnarsson S., Kahl J., Kesse-Guyot E., Rembiałkowska E., Quaglio G., Grandjean P. (2017). Human health implications of organic food and organic agriculture: A comprehensive review. Environ. Health Glob. Access. Sci. Source.

[3] Baudry J., Assmann K.E., Touvier M., Allès B., Seconda L., Latino-Martel P., Ezzedine K., Galan P., Hercberg S., Lairon D. (2018). Association of Frequency of Organic Food Consumption with Cancer Risk: Findings from the NutriNet-Santé Prospective Cohort Study. JAMA Intern. Med..

[4] Santé publique France Recommandations Relatives à L’alimentation, à L’activité Physique et à la Sédentarité pour les Adultes. Saint-Maurice, France, 2017. https://static.cnsf.asso.fr/wp-content/uploads/2019/01/2019_SPF_reco-alimentation-activite-physique-sedentarite-adultes.pdf.

[5] French Agency for the Development and Promotion of Organic Agriculture (2018). L’agriculture Biologique, un Accélérateur Économique, à la Résonnance Sociale et Sociétale. https://www.agencebio.org/wp-content/uploads/2018/10/dossierdepressechiffres-juin2018.pdf.

[6] Baudry J., Péneau S., Allès B., Touvier M., Hercberg S., Galan P., Amiot M.J., Lairon D., Méjean C., Kesse-Guyot E. (2017). Food Choice Motives When Purchasing in Organic and Conventional Consumer Clusters: Focus on Sustainable Concerns (The NutriNet-Santé Cohort Study). Nutrients.

[7] Péneau S., Ménard E., Méjean C., Bellisle F., Hercberg S. (2013). Sex and dieting modify the association between emotional eating and weight status. Am. J. Clin. Nutr..

[8] Julia C., Péneau S., Andreeva V.A., Méjean C., Fezeu L., Galan P., Hercberg S. (2014). Weight-loss strategies used by the general population: How are they perceived?. PLoS ONE.

[9] Santos I., Sniehotta F.F., Marques M.M., Carraça E.V., Teixeira P.J. (2017). Prevalence of personal weight control attempts in adults: A systematic review and meta-analysis. Obes. Rev..

[10] Schaumberg K., Anderson D.A., Anderson L.M., Reilly E.E., Gorrell S. (2016). Dietary restraint: What’s the harm? A review of the relationship between dietary restraint, weight trajectory and the development of eating pathology. Clin. Obes..

[11] Lowe M.R., Doshi S.D., Katterman S.N., Feig E.H. (2013). Dieting and restrained eating as prospective predictors of weight gain. Front. Psychol..

[12] Stunkard A.J., Messick S. (1985). The three-factor eating questionnaire to measure dietary restraint, disinhibition and hunger. J. Psychosom. Res..

[13] De Lauzon B., Romon M., Deschamps V., Lafay L., Borys J.M., Karlsson J., Ducimetière P., Charles M.A. (2004). Fleurbaix Laventie Ville Sante Study Group. The Three-Factor Eating Questionnaire-R18 Is Able to Distinguish among Different Eating Patterns in a General Population. J. Nutr..

[14] De Castro J.M. (1995). The relationship of cognitive restraint to the spontaneous food and fluid intake of free-living humans. Physiol. Behav..

[15] Rideout C.A., McLean J.A., Barr S.I. (2004). Women with high scores for cognitive dietary restraint choose foods lower in fat and energy. J. Am. Diet. Assoc..

[16] Anschutz D.J., Van Strien T., Van De Ven M.O.M., Engels R.C.M.E. (2009). Eating styles and energy intake in young women. Appetite.

[17] Tuschl R.J., Laessle R.G., Platte P., Pirke K.M. (1990). Differences in food-choice frequencies between restrained and unrestrained eaters. Appetite.

[18] Richardson A.S., Arsenault J.E., Cates S.C., Muth M.K. (2015). Perceived stress, unhealthy eating behaviors, and severe obesity in low-income women. Nutr. J..

[19] Nogay N.H. (2017). The role of psychological eating styles in obesity among Turkish adolescents: A cross-sectional study. JPMA J. Pak. Med. Assoc..

[20] Teixeira P.J., Silva M.N., Coutinho S.R., Palmeira A.L., Mata J., Vieira P.N., Carraça E.V., Santos T.C., Sardinha L.B. (2010). Mediators of weight loss and weight loss maintenance in middle-aged women. Obesity.

[21] Urbanek J.K., Metzgar C.J., Hsiao P.Y., Piehowski K.E., Nickols-Richardson S.M. (2015). Increase in cognitive eating restraint predicts weight loss and change in other anthropometric measurements in overweight/obese premenopausal women. Appetite.

[22] Singh A., Bains K., Kaur H. (2017). Relationship of Eating Behaviors with Age, Anthropometric Measurements, and Body Composition Parameters among Professional Indian Women. Ecol. Food Nutr..

[23] Provencher V., Drapeau V., Tremblay A., Després J.-P., Lemieux S. (2003). Eating behaviors and indexes of body composition in men and women from the Québec family study. Obes. Res..

[24] De Lauzon-Guillain B., Basdevant A., Romon M., Karlsson J., Borys J.M., Charles M.A. (2006). FLVS Study Group. Is restrained eating a risk factor for weight gain in a general population?. Am. J. Clin. Nutr..

[25] Drapeau V., Provencher V., Lemieux S., Després J.-P., Bouchard C., Tremblay A. (2003). Do 6-y changes in eating behaviors predict changes in body weight? Results from the Québec Family Study. Int. J. Obes. Relat. Metab. Disord..

[26] Kruger J., Galuska D.A., Serdula M.K., Jones D.A. (2004). Attempting to lose weight: Specific practices among U.S. adults. Am. J. Prev. Med..

[27] Hercberg S., Castetbon K., Czernichow S., Malon A., Mejean C., Kesse E., Touvier M., Galan P. (2010). The Nutrinet-Santé Study: A web-based prospective study on the relationship between nutrition and health and determinants of dietary patterns and nutritional status. BMC Public Health.

[28] Tholin S., Rasmussen F., Tynelius P., Karlsson J. (2005). Genetic and environmental influences on eating behavior: The Swedish Young Male Twins Study. Am. J. Clin. Nutr..

[29] Kesse-Guyot E., Castetbon K., Touvier M., Hercberg S., Galan P. (2010). Relative validity and reproducibility of a food frequency questionnaire designed for French adults. Ann. Nutr. Metab..

[30] Baudry J., Méjean C., Allès B., Péneau S., Touvier M., Hercberg S., Lairon D., Galan P., Kesse-Guyot E. (2015). Contribution of Organic Food to the Diet in a Large Sample of French Adults (the NutriNet-Santé Cohort Study). Nutrients.

[31] Schofield W.N. (1985). Predicting basal metabolic rate, new standards and review of previous work. Hum. Nutr. Clin. Nutr..

[32] Institut National de la Statistique et des Études Économiques–INSEE Définition-Consumption Unit|Insee. http://www.insee.fr/en/metadonnees/definition/c1802.

[33] Craig C.L., Marshall A.L., Sjöström M., Bauman A.E., Booth M.L., Ainsworth B.E., Pratt M., Ekelund U., Yngve A., Sallis J.F. (2003). International physical activity questionnaire: 12-country reliability and validity. Med. Sci. Sports Exerc..

[34] World Health Organization (2000). Obesity: Preventing and Managing the Global Epidemic.

[35] Estaquio C., Kesse-Guyot E., Deschamps V., Bertrais S., Dauchet L., Galan P., Hercberg S., Castetbon K. (2009). Adherence to the French Programme National Nutrition Santé Guideline Score is associated with better nutrient intake and nutritional status. J. Am. Diet. Assoc..

[36] Bénard M., Bellisle F., Etilé F., Reach G., Kesse-Guyot E., Hercberg S., Péneau S. (2018). Impulsivity and consideration of future consequences as moderators of the association between emotional eating and body weight status. Int. J. Behav. Nutr. Phys. Act..

[37] Lattimore P., Fisher N., Malinowski P. (2011). A cross-sectional investigation of trait disinhibition and its association with mindfulness and impulsivity. Appetite.

[38] Petersen S.B., Rasmussen M.A., Strøm M., Halldorsson T.I., Olsen S.F. (2013). Sociodemographic characteristics and food habits of organic consumers--a study from the Danish National Birth Cohort. Public Health Nutr..

[39] Torjusen H., Brantsæter A.L., Haugen M., Alexander J., Bakketeig L.S., Lieblein G., Stigum H., Næs T., Swartz J., Holmboe-Ottesen G. (2014). Reduced risk of pre-eclampsia with organic vegetable consumption: Results from the prospective Norwegian Mother and Child Cohort Study. BMJ Open.

[40] Eisinger-Watzl M., Wittig F.A., Heuer T., Hoffmann I. (2015). Customers Purchasing Organic food-do they live Healthier? Results of the German National Nutrition Survey II. Eur. J. Nutr. Food Saf..

[41] Nicklas J.M., Huskey K.W., Davis R.B., Wee C.C. (2012). Successful weight loss among obese U.S. adults. Am. J. Prev. Med..

[42] Foster C., Padel S. (2005). Exploring the gap between attitudes and behaviour: Understanding why consumers buy or do not buy organic food. Br. Food J..

[43] Provencher V., Bégin C., Gagnon-Girouard M.-P., Tremblay A., Boivin S., Lemieux S. (2008). Personality traits in overweight and obese women: Associations with BMI and eating behaviors. Eat Behav..

[44] Keller C., Siegrist M. (2015). Does personality influence eating styles and food choices? Direct and indirect effects. Appetite.

[45] Longnecker M.P., Harper J.M., Kim S. (1997). Eating frequency in the Nationwide Food Consumption Survey (U.S.A.), 1987–1988. Appetite.

[46] Park M.K., Freisling H., Huseinovic E., Winkvist A., Huybrechts I., Crispim S.P., de Vries J.H.M., Geelen A., Niekerk M., van Rossum C. (2018). Comparison of meal patterns across five European countries using standardized 24-h recall (GloboDiet) data from the EFCOVAL project. Eur. J. Nutr..

[47] Sobal J., Bisogni C.A. (2009). Constructing food choice decisions. Ann. Behav. Med..

[48] Zanoli R., Naspetti S. (2002). Consumer motivations in the purchase of organic food: A means-end approach. Br. Food J..

[49] Yadav R., Pathak G.S. (2016). Intention to purchase organic food among young consumers: Evidences from a developing nation. Appetite.

[50] Harper G.C., Makatouni A. (2002). Consumer perception of organic food production and farm animal welfare. Br. Food J..

[51] Baudry J., Méjean C., Péneau S., Galan P., Hercberg S., Lairon D., Kesse-Guyot E. (2015). Health and dietary traits of organic food consumers: Results from the NutriNet-Santé study. Br. J. Nutr..

[52] Grunert K.G., Wills J.M. (2007). A review of European research on consumer response to nutrition information on food labels. J. Public Health.

[53] Blake C.E., Bisogni C.A., Sobal J., Jastran M., Devine C.M. (2008). How adults construct evening meals. Scripts for food choice. Appetite.

[54] Jastran M.M., Bisogni C.A., Sobal J., Blake C., Devine C.M. (2009). Eating routines. Embedded, value based, modifiable, and reflective. Appetite.

[55] Engler-Stringer R. (2011). Food selection and preparation practices in a group of young low-income women in Montreal. Appetite.

[56] French S.A. (2003). Pricing effects on food choices. J. Nutr..

[57] Jetter K.M., Cassady D.L. (2006). The availability and cost of healthier food alternatives. Am. J. Prev. Med..

[58] Monsivais P., Drewnowski A. (2007). The rising cost of low-energy-density foods. J. Am. Diet. Assoc..

[59] Food and Agriculture Organization (FAO) (2016). Organic Agriculture: Why Is Organic Food More Expensive Than Conventional Food?. http://www.fao.org/organicag/oa-faq/oa-faq5/en/.

[60] Grogan S. (2006). Body image and health: Contemporary perspectives. J. Health Psychol..

[61] Gilg A., Barr S., Ford N. (2005). Green consumption or sustainable lifestyles? Identifying the sustainable consumer. Futures.

[62] Panzone L., Hilton D., Sale L., Cohen D. (2016). Socio-demographics, implicit attitudes, explicit attitudes, and sustainable consumption in supermarket shopping. J. Econ. Psychol..

[63] Bender R., Lange S. (2001). Adjusting for multiple testing—When and how?. J. Clin. Epidemiol..

[64] Shim J.-S., Oh K., Kim H.C. (2014). Dietary assessment methods in epidemiologic studies. Epidemiol. Health.

[65] Svensson M., Hult M., van der Mark M., Grotta A., Jonasson J., von Hausswolff-Juhlin Y., Rössner S., Trolle Lagerros Y. (2014). The change in eating behaviors in a Web-based weight loss program: A longitudinal analysis of study completers. J. Med. Internet Res..

[66] Spencer E.A., Appleby P.N., Davey G.K., Key T.J. (2002). Validity of self-reported height and weight in 4808 EPIC-Oxford participants. Public Health Nutr..

[67] Lassale C., Péneau S., Touvier M., Julia C., Galan P., Hercberg S., Kesse-Guyot E. (2013). Validity of web-based self-reported weight and height: Results of the Nutrinet-Santé study. J. Med. Internet Res..

