# Supplementary material for: Cognitive Restraint and History of Dieting Are Negatively Associated with Organic Food Consumption in a Large Population-Based Sample of Organic Food Consumers
Source: Nutrients. 2019 Oct 15;11(10):2468. doi: 10.3390/nu11102468 (PMC6836016; doi:10.3390/nu11102468)
Supplement: Supplementary file 1 [file nutrients-11-02468-s001.pdf]

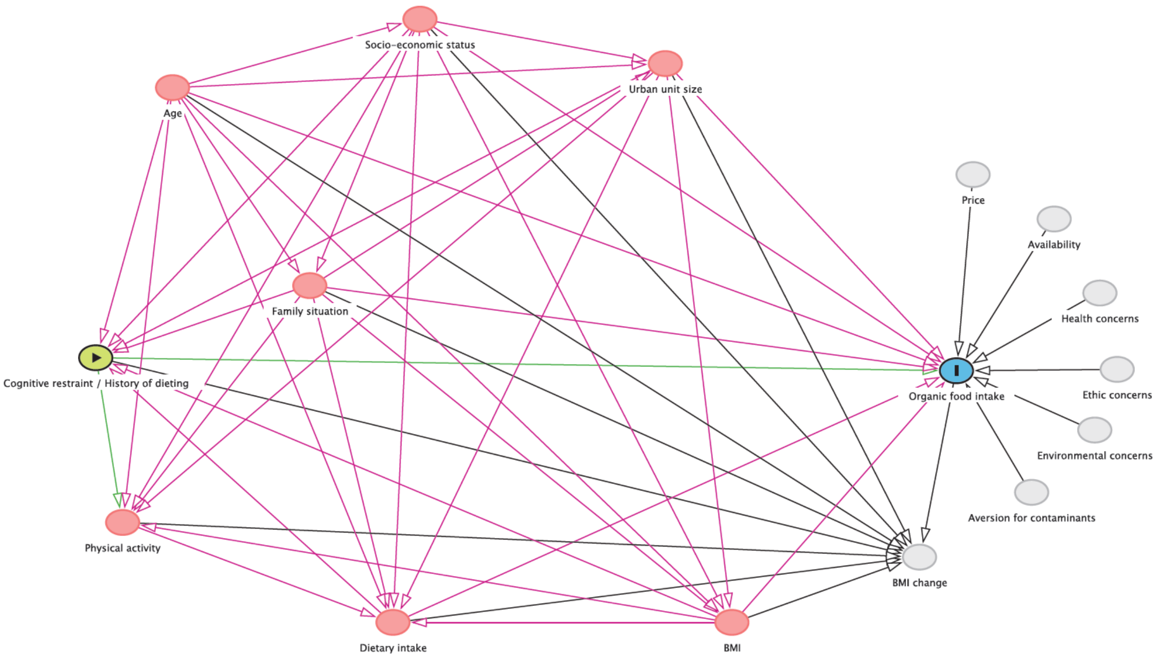

Figure S1. Directed acyclic graph (DAG) representing associations between cognitive restrain/history of dieting and organic food consumption. Minimal sufficient adjustment sets for estimating the total effect of cognitive restrain/history of dieting on organic food intake as suggested by the DAG are: age, BMI, dietary intake, family situation, socioeconomic status, urban unit size. Colors of variables: green—exposure; blue—outcome; red—co-variables. (NutriNet-Santé study (2010–2014).
